# Miracles, Martyrdom and Violence: Historical Origins of the Patron Saints of Trauma and Orthopaedic Surgery

**DOI:** 10.7759/cureus.21355

**Published:** 2022-01-18

**Authors:** John T Williams, Eleanor R Kissin, Mark W Kissin

**Affiliations:** 1 Trauma and Orthopaedics, King's College Hospital, London, GBR; 2 Trauma and Orthopaedics, Lewisham Hospital, London, GBR; 3 Breast and Melanoma Surgery, Retired, Guildford, GBR

**Keywords:** surgical history, christianity, religion, trauma, orthopaedics, orthopaedic history

## Abstract

Patron saints have been adopted to protect against disease and disability in the Christian world since the Middle Ages. A patron saint most often has a morbid connection to their affiliated ailment, although patronage may stem from physical attributes or a miracle performed. The purpose of this study is to identify and describe patron saints associated with injury, musculoskeletal disease and orthopaedic pathology. Saints were identified by a systematic review of multiple reference texts. Additional searches were performed in online academic databases, alongside biographic research of primary and secondary archives.

Seven patron saints associated with trauma and orthopaedic conditions were identified. These include St. Ignatius, who pioneered deformity-correction surgery on himself and St. Kostka, patron saint of broken bones who was the victim of his infamously cruel brother Paul. St. Alphonsus Liguori, patron saint of spinal conditions, suffered such a severe cervical kyphosis that his chin eroded his chest. Further saints identified include St. Cosmas and St. Damian as patrons of musculoskeletal oncology, and St. Amalberga and St. Roch as patrons of upper and lower limb injuries, respectively. Over the centuries, patron saints have provided hope for patients in the absence of effective treatments, and as role models for physicians with few resources. Their lives and legends provide valuable insight into an important historical aspect of medical culture.

## Introduction and background

Patron saints to protect against disease and disability have been adopted throughout the Christian world since the Middle Ages. This phenomenon was particularly prevalent within disadvantaged populations in Europe, who had little access to doctors or medicine [1,2]. Patron saints of disease represent an important and unique aspect of medical history and culture, which is important to acknowledge and preserve in contemporary scientific literature. A small number of notable saints have been previously explored in a modern medical context [1,3-5]; however, the patron saints of trauma and orthopaedic surgery have not been previously collectively described.

A saint most often became associated with a particular disorder as a result of their method of martyrdom. Perhaps the most well-known example is St. Agatha’s patronage of breast cancer, conferred as a result of her martyrdom by double mastectomy in 251 CE by the Roman Governor Quintianus [4,6]. Alternatively, patronage may stem from notable physical attributes or a certain miracle performed. 

There are more than 300 recognised patron saints in medicine and of specific diseases, although overlap and contradiction exist between sources [7-9]. Hagiographies are notoriously enigmatic sources for academics, with multiple authors over the centuries conflating and aggrandising accounts of their subjects' lives. Saintly patronage is generally acknowledged on the basis of longstanding tradition and custom and there is often considerable obscurity regarding the exact origin of the link between an ailment and a saint. 

## Review

Methods

A systematic review of online editions of four standard reference texts on saints [2,7,8] was performed by authors JW and EK between January and March 2021, using key terms for anatomic locations (arm, shoulder, elbow, hand, leg, hip, knee, foot, spine), injuries (broken bones, accidents, fractures, wounds) and orthopaedic conditions (arthritis, back pain, limb/joint pain, surgery, hip/knee replacement, rheumatism). Further searches were performed in online databases (PubMed, Medline, Cochrane Library, ScienceDirect, Google Books) using the same search terms. Reference lists in relevant studies were examined for additional sources. Further relevant patron saints encountered by the authors in secondary sources were included.

Inclusion criteria were defined as an individual canonised or otherwise recognised as a saint by the Roman Catholic Church, with documented patronage of a musculoskeletal complaint, injury or other orthopaedic condition. The resultant list of candidates was screened for overlap, repetition and contradiction. Saintly patronage that was supported by multiple sources, with the fewest contradictions, was deemed suitable for inclusion in the study. Saints are frequently patrons of a large number of causes, so in the case of overlap, the more prominent advocate was selected. Subsequent further biographic research using primary and secondary archives and documents was then undertaken.

Results

Seven saints associated with trauma and orthopaedic surgery were identified for inclusion in the study, spanning 270 CE - 1787 CE. We present the lives, legends and pictorial representations of each saint in chronological order. 

St. Cosmas and St. Damian - Patron Saints of Musculoskeletal Oncology

The first candidates are a much-celebrated surgical partnership: the twins Cosmas and Damian (born {b.} 270 CE, Arabia). Both studied medicine in the ancient Greek city of Antioch before returning to practice in their hometown of Aegeae, a small port situated in what is now modern-day Turkey. The twins were famous for their medical prowess yet refused payment for their services, earning the name “the silverless ones” or anargyroi in Greek [3,10].

**Figure 1:**
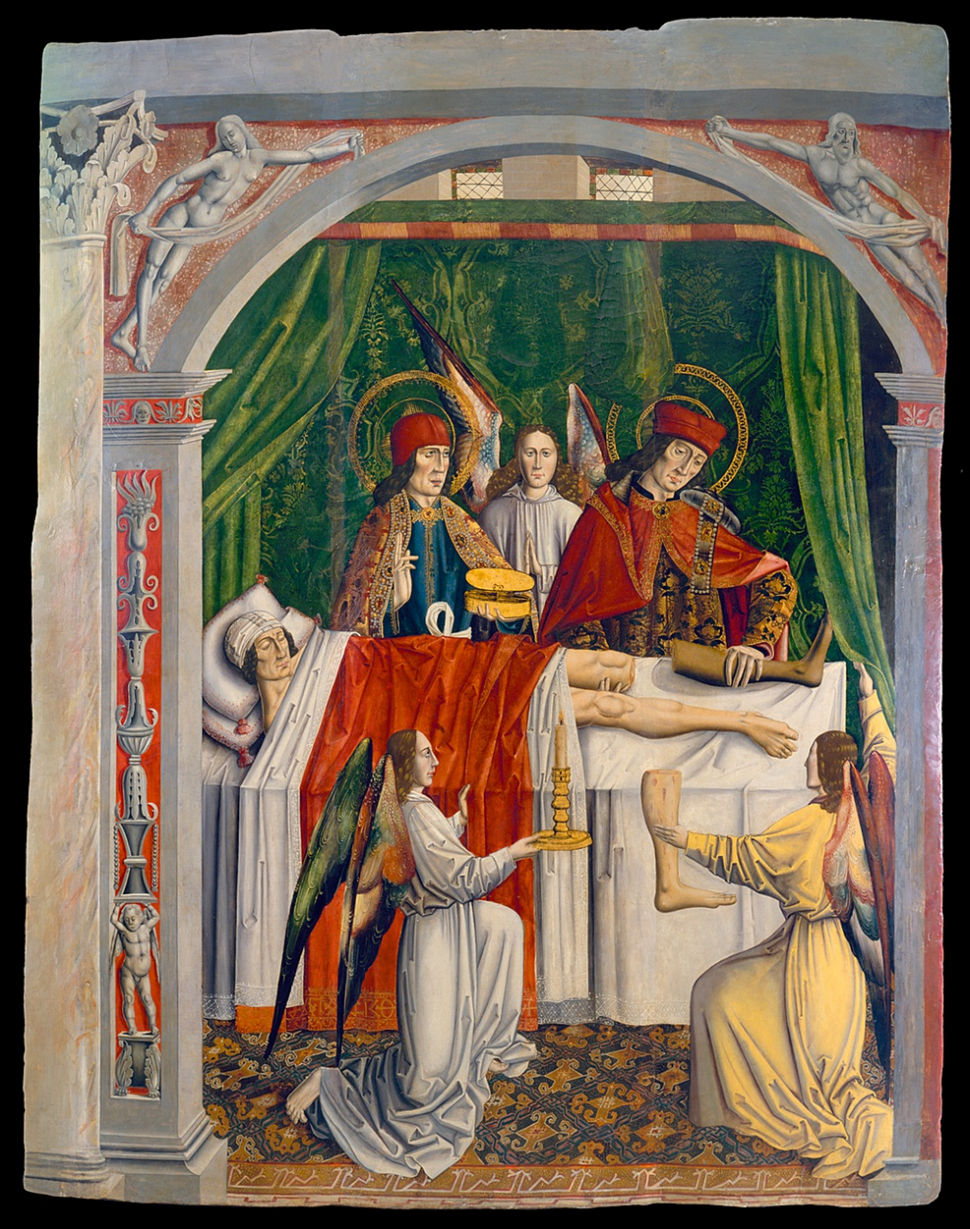
A verger’s dream: Saints Cosmas and Damian performing a miraculous leg transplantation "A verger's dream: Saints Cosmas and Damian performing a miraculous cure by transplantation of a leg. Oil painting attributed to the Master of Los Balbases, ca. 1495. Permission under  Public Domain Mark," ed: Wellcome Collection.

Their most remarkable feat is vividly depicted in numerous artworks and has inspired a number of articles in modern medical literature (Figure 1). The saints are credited with having performed what appears to be the first homoplastic limb transplant, for a presumed sarcoma of the leg [11]. The recipient in question was Deacon Justinian of Rome, who was suffering from what has been variously described as cancer, tumour or “canker” of the leg according to Jacopo da Varagine’s 13th century Legenda Aurea [12,13]. The cadaveric donor was an Ethiopian patient who had died the previous day, giving rise to the feat becoming popularised as the Miracle of the Black Leg. The Deacon was reported to subsequently have made a full recovery.

The Saints’ continued devotion to the Christian faith, unfortunately, attracted the ire of the Roman emperor of the day Diocletian, who ordered the twins imprisoned, tortured and eventually beheaded in 303 CE. Their feast day falls on September 27th.

St. Amalberga of Temse - Patron Saint of the Upper Limb

St. Amalberga of Temse (b. 741 CE, Ardennes, Belgium) is commonly invoked as the patron saint of pain, fractures, surgery and injuries of the arm, due to the legend of the assault inflicted upon her by Charlemagne, or Charles the Great, in Meteren, Netherlands.

Amalberga was renowned throughout mid-eighth century Western Europe for her remarkable beauty, nobility and devoutness. Her reputation attracted the attention of Pepin III, King of the Franks, who insisted she marry his eldest son, Charlemagne [14]. Amalberga, at that time a ward of the Abbess Landrada at the nunnery in Munsterbilzen, declined his proposal. Charlemagne himself came to the nunnery to persuade her but to no avail. Described in the 12th century Historia Caroli Magni, or The Pseudo-Turpin Chronicle, in an unusual and fortuitous distraction, Charlemagne became embroiled in a bear hunt which allowed Amalberga time to escape his advances [15]. King Pepin became tired of waiting for his son to find her and married him to another.

Not to be deterred, Charlemagne eventually traced her whereabouts to a church in Meteren but was horrified to find she had cut off her hair in an attempt to disguise herself from him. In a fit of rage, he infamously fractured her arm attempting to drag her from the church. Miraculously, the limb healed instantaneously and she managed to escape him again to the banks of the Scheld river [7]. An enormous sturgeon, with which she is usually depicted, was said to have risen out of the water and carried her on its back to safety in Temse, where her relics and chapel can be found today. She died in Temse in 772 CE and her feast day is 10th July.

St. Roch - Patron Saint of the Knee (and Lower Limb)

**Figure 2:**
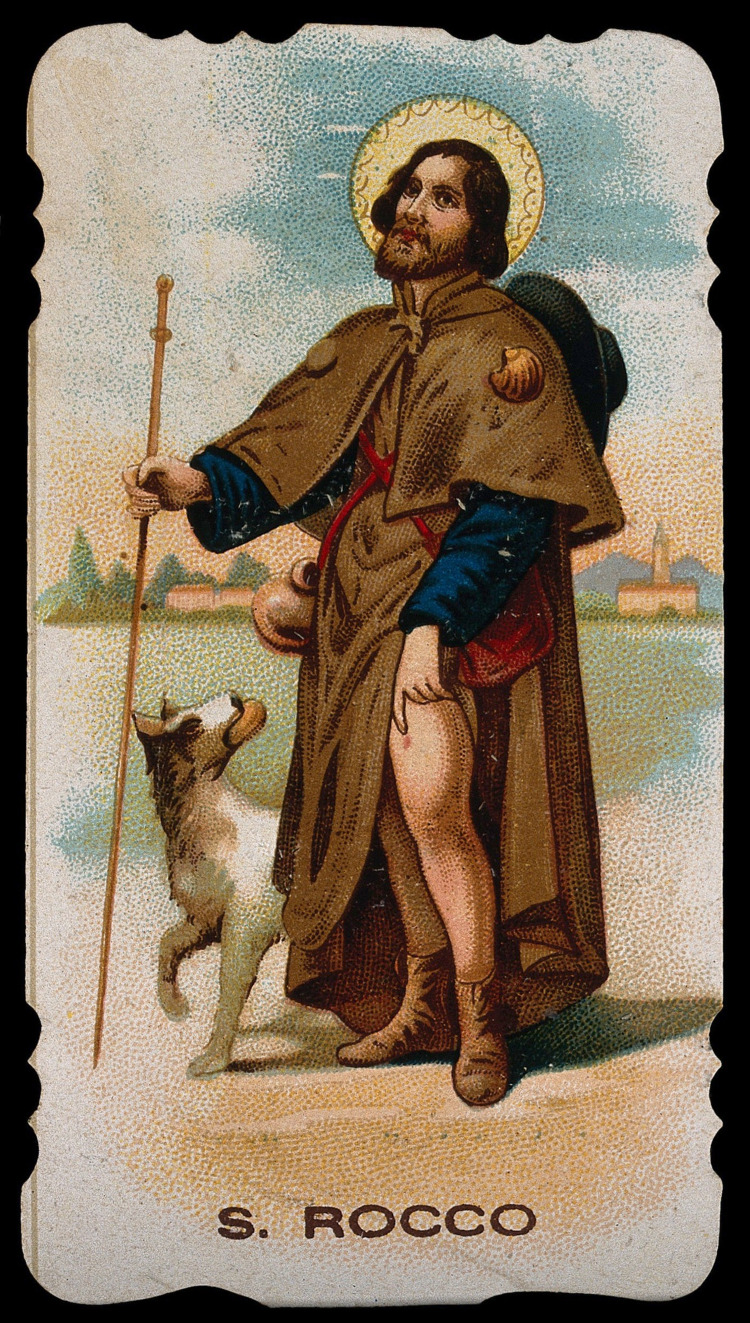
Colour lithograph of St. Roch (or Rocco) Colour lithograph of St. Roch (or Rocco) demonstrating the lesion on his left leg, unknown artist, 1906. Credit:  Wellcome Collection. Permission under  Attribution 4.0 International (CC BY 4.0); URL: https://wellcomecollection.org/works/h7e8tu58," ed: Wellcome Collection.

St. Roch (b. 1348 Montpellier, France) is widely venerated in Europe and commonly invoked as the patron saint of knee complaints, in addition to epidemics, skin infections and dogs [8]. His patronage of the knee stems from his representation in Christian iconography, which traditionally depicts the saint standing with one leg exposed to the thigh, knee slightly flexed, indicating to the limb (Figure 2).

Son and heir to the noble Governor of Montpellier, he nonetheless gave away all his material possessions to the poor and began a pilgrimage to Rome [2]. The bubonic plague was gripping Italy in the mid-14th century and moving Northwards into the rest of Europe [16]. St. Roch devoted himself to tending the victims of the Black Death but unfortunately succumbed to the infection himself. He retreated to the woods to die but was miraculously saved by a local hunting dog who brought him water and, as often represented, licked the bubonic lesions on his leg until they healed. Sadly, he later died anonymously in jail having been falsely arrested as a spy by his own uncle in a calamitous case of mistaken identity. He was canonised in 1427 and his feast day is celebrated on August 16th.

St. Ignatius of Loyola - Patron Saint of Trauma and Fracture Surgery

**Figure 3:**
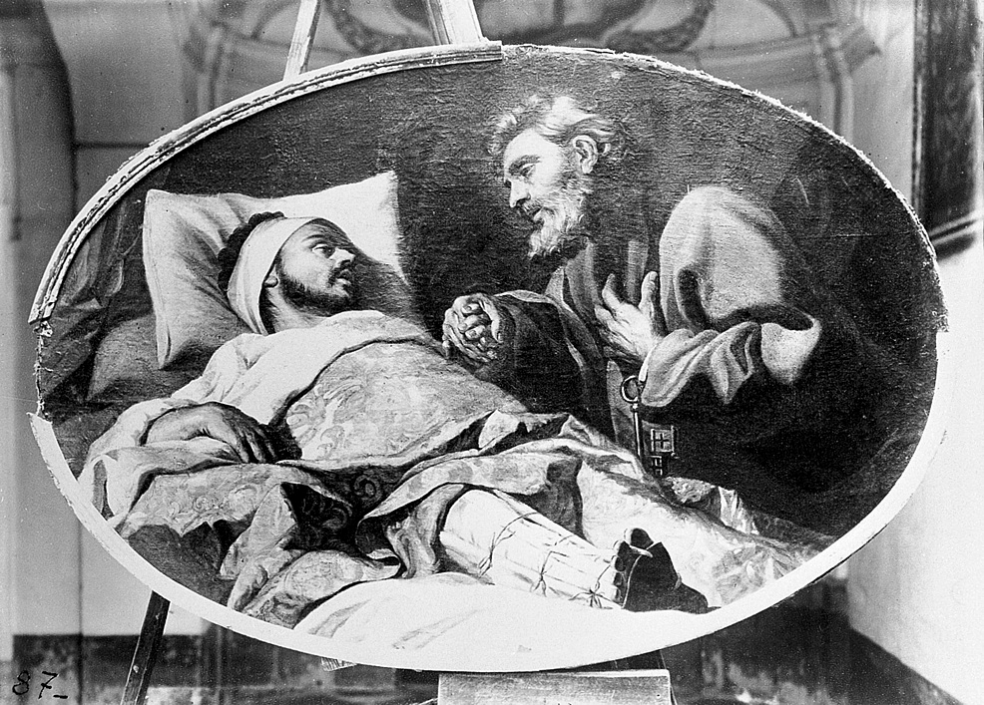
St. Ignatius Loyola wearing leg splints St. Ignatius Loyola wearing leg splints has a vision of St. Peter; by De Favray, in the Archbishop’s Curia, Floriana, Malta. Credit:  Wellcome Collection.  Permission under Attribution 4.0 International (Creative Commons BY 4.0); URL:  https://wellcomecollection.org/works/raa4976u," ed: Wellcome Collection.

St. Ignatius (b. 1491, Loyola, Spain) spent his early life as a knight in the service of Antonio Manrique de Lara, 2nd Duke of Nájera. Whilst defending the town of Pamplona against attacking French forces, he suffered a devastating cannonball injury to both lower limbs, sustaining bilateral femoral fractures (Figure 3). Fortunately, he survived the initial trauma but was left with a significant leg length discrepancy and an unsightly limb deformity [17,18]. 

Discontented, he underwent a series of procedures to ‘re-set’ the fractures. This included a prolonged period of time with his leg on traction, counter-weighted with a cannonball, in an attempt to restore anatomic alignment [19]. Whilst the functional outcome was inadequate to resume his career as a soldier, an unexpected vocation presented itself during his convalescence. Only two books were available for him to read: one on Jesus’ life, and another on the exploits of the saints. He was so moved by the texts that he renounced his military career and eventually became a priest in Rome, later founding the Jesuit order. He was canonised in 1622 and his feast day falls on 31st July. 

St. Stanislaus Kostka - Patron Saint of Broken Bones

St. Stanislaus Kostka (b. 1550, Poland) is commonly invoked as the patron saint of broken bones. He was the second of seven children born into a noble and wealthy family in Rostkowo, Poland. Aged fourteen, Stanislaus and his elder brother Paul were sent to Vienna to attend Jesuit College. During his schooling there, Stanislaus became a particularly pious and devout child which made him unhappily conspicuous amongst his fellow students. He was said to have often fallen unconscious in the church of Jesuit Fathers, such was his devotion [20]. 

**Figure 4:**
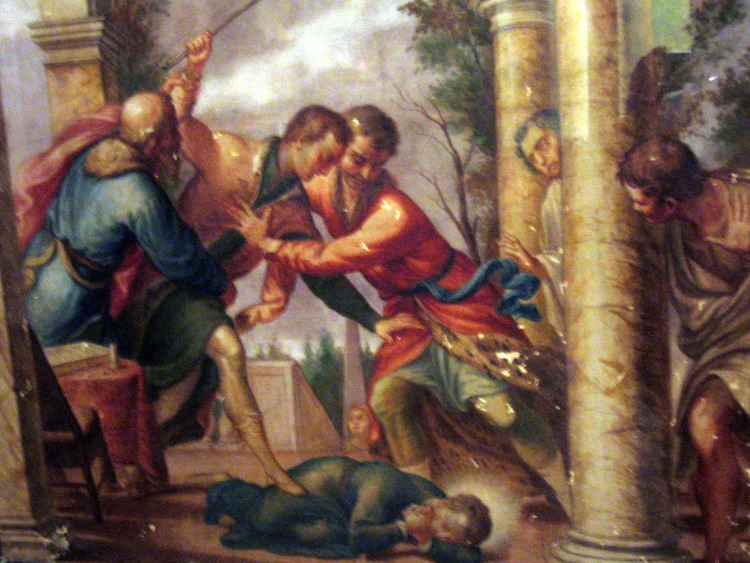
St. Stanislaus Kostka being brutally beaten by his brother Paul "St. Stanislaus Kostka is beaten by his brother Paul; by Andrea Pozzo, in Basilica dei Santi Apostoli (Rome). Permission under Attribution 2.0 Generic Creative Commons (photograph Anthony Majanlahti 2005); URL:  https://www.flickr.com/people/93226994@N00," ed: Wikimedia Commons.

Stanislaus’ relationship with his brother Paul was infamously violent. Paul would become so enraged at his brother’s religious fervour that he subjected Stanislaus to violent attacks (Figure 4) resulting in the multiple fractures of which he is now patron [21]. His recovery was often slow and protracted, although he eventually managed to flee Vienna to Rome to join the Jesuit Society there. He remained a mirror of religious perfection, although died after only ten months in Rome, aged seventeen. He was canonised in 1726 and his feast day is November 13th.

St. Alphonsus of Liguori - Patron Saint of Spinal Conditions

St. Alphonsus (b. 1696, Naples, Italy) studied Law at the University of Naples and spent his early working life as a successful lawyer [2]. He later became disillusioned and the loss of a difficult case led Alphonsus to abandon the legal profession and become a priest. He had an illustrious career in the Roman church and was a renowned theologian, orator, musician and scholar. He was however plagued by ill health throughout his life, remarkably receiving the last rites on numerous occasions in 1726, 1762 and 1768, before his eventual death aged 90 in 1787. 

His patronage of spinal disorders arose from an affliction that tormented him in later years. He developed a progressive cervical kyphosis so severe his chin eroded into his anterior chest wall [22]. Alphonsus also suffered severe sciatica (“his constant companion”), paraparesis and was frequently bedridden. Despite this, he continued his duties as a priest and published a number of major contributions to canon law and theology. McLauglin et al. published a study on photographs and radiographs of the Saint’s osseous remains, concluding he likely suffered both Paget’s disease and tuberculous osteomyelitis of the spine [23]. He was canonised in 1839 and his feast day is 1st August.

## Conclusions

Over the centuries, patron saints have provided a source of comfort for patients in the absence of effective treatments, and as role models for physicians with few resources. The lives and legends of the patron saints described may be far removed from contemporary practice, but nonetheless provide an interesting and valuable insight into a unique aspect of medicine in Christian culture and history. It is important to record and preserve these accounts in contemporary scientific literature. Patron saints are likely to continue to serve as a source of inspiration, hope and consolation to certain groups of patients, and indeed colleagues, for years to come.
